# Institutional factors associated with early mortality of newly diagnosed acute promyelocytic leukemia

**DOI:** 10.1038/s41408-022-00767-6

**Published:** 2022-12-15

**Authors:** Kensuke Matsuda, Taisuke Jo, Hiroki Matsui, Kiyohide Fushimi, Hideo Yasunaga, Koichi Sugimoto

**Affiliations:** 1 Department of Hematology and Oncology, JR Tokyo General Hospital, Tokyo, Japan; 2 Department of Health Services Research, Graduate School of Medicine, The University of Tokyo, Tokyo, Japan; 3 Department of Clinical Epidemiology and Health Economics, School of Public Health, The University of Tokyo, Tokyo, Japan; 4 Department of Health Policy and Informatics, Tokyo Medical and Dental University Graduate School of Medicine, Tokyo, Japan

**Keywords:** Acute myeloid leukaemia, Risk factors

**Dear Editor**,

Acute promyelocytic leukemia (APL) is a clinical entity characterized by a translocation between chromosomes 15 and 17 [1, 2]. All-trans retinoic acid (ATRA) has been found to markedly improve prognosis, and previous clinical trials have shown that the complete remission rate reaches ~90% [2–5]. However, previous real-world studies showed that early mortality was still high in unselected APL populations [6–8]. Our nationwide study also demonstrated that although all patients received ATRA, early mortality reached 16% in a real-world clinical setting in Japan [9]. The present study aimed to assess the association between institutional factors and early death during induction therapy for untreated APL, using a Japanese nationwide inpatient database.

This was a retrospective cohort study using the Diagnosis Procedure Combination database, a nationwide inpatient database in Japan. The database includes discharge abstracts and administrative claims data for >1200 acute-care hospitals and covers approximately 90% of all tertiary-care emergency hospitals in Japan [10], and has been used in various nationwide real-world studies [11]. The database includes data on patient age, sex, body height, body weight, Barthel index [12], comorbidities at admission, post-admission complications, volume of transfusion, drug dosage, duration of drug administration, length of stay, medical costs during hospitalization, and discharge status. Primary diagnosis, comorbidities, and complications were recorded with the International Classification of Diseases and Related Health Problems, Tenth Revision (ICD-10) codes. This study was approved by the Ethics Review Board of The University of Tokyo and was conducted in accordance with the principles of the Declaration of Helsinki. Patients who were hospitalized for the treatment of APL (ICD-10 code: C92.4) between July 2010 and March 2018 were evaluated. Hospitalizations in which induction therapy including ATRA was initiated for patients with untreated APL were selected. In addition, because >70% of patients with APL were reported to develop coagulopathy [13], we further selected patients who required fresh frozen plasma (FFP) transfusion within 3 days of admission to more accurately identify APL patients from the database. This definition was also used in our previous studies [9, 14]. Patient covariates included age, sex, body mass index (BMI), activities of daily living, Charlson comorbidity index (CCI) [15], initial FFP transfusion volume (i.e., initial transfusion volume of FFP per body weight within 3 days of admission), and emergency admission. BMI (kg/m^2^) was divided into <18.5, 18.5–24.9, 25.0–29.9, ≥30.0, or missing. Activities of daily living were evaluated using the Barthel index [12], with being fit defined as the maximum score of the Barthel index. The CCI was scored using diagnoses for individual patients and was categorized into 2 or ≥3. Initial FFP transfusion volume (ml/kg) was categorized into three groups and used as a proxy indicator for the severity of coagulopathy [9]. In addition to these patients’ baseline factors, we added the following institutional covariates: hospital volume, location of hospital, educational hospital, the number of beds in each hospital, and the number of hematologists per population in each prefecture. The hospital volume was divided into two categories. Hospitals that treated >5 newly diagnosed APL patients hospitalized during the study period were defined as high-volume hospitals, and the others were defined as low-volume hospitals. These definitions were based on the median value of the number of treated patients during the relevant period (median 5, interquartile range 3–8). Hospitals were also divided into urban and non-urban areas. Major cities (the 23 wards of Tokyo and the 20 ordinance-designated cities) were defined as urban areas. Ordinance-designated cities have a population greater than 500,000. The numbers of beds were divided into ≥400 and <400. The number of hematologists in each prefecture was obtained from The Japanese Society of Hematology. The number of hematologists per 100,000 people in each prefecture was estimated based on the data in 2014, the median year of the study period, and 47 prefectures were divided equally into four categories: tier 1: ≥3.200 (12 prefectures), tier 2: ≥2.464, <3.200 (12 prefectures); tier 3: ≥1.950, <2.464 (11 prefectures), and tier 4, <1.950 (12 prefectures). The primary outcome was all-cause in-hospital mortality during hospitalization. A multivariable logistic regression model was used to evaluate risk factors for the primary outcome. Generalized estimating equations were fitted using the multivariable logistic regression model to adjust for within-hospital clustering. Independent variables included patient characteristics (age, sex, body mass index, comorbidity index, activity of daily living, initial FFP transfusion volume, and emergency admission) and institutional factors (hospital volume, location of hospital, educational hospital, the number of beds in each hospital, and the number of hematologists per population in each prefecture). The association between the institutional factors and treatment practices was further analyzed using univariate analyses by the chi-square test. Regarding treatment practices, immediate initiation of ATRA was defined as ATRA administration within 1 day from admission, and prompt initiation of conventional chemotherapy was defined as the start within 7 days from admission. All statistical analyses were conducted with SPSS, Version 25.0 (IBM SPSS, Armonk, NY). The level of significance was defined as a two-tailed *P* < 0.05.

A total of 1138 patients who received induction therapy including ATRA were identified. Institutional and patient characteristics in the overall cohort are shown in Table 1. Overall, 461 (41%) patients were treated at high-volume hospitals. The number of hematologists per population in each prefecture is described in Supplemental Table 1. There were 195 (17%) patients who died within a median of 11 days of hospitalization (interquartile range, 5–25 days). In the multivariable model, treatment at high-volume hospitals was significantly associated with lower in-hospital mortality (odds ratio 0.60 [95% confidence interval: 0.42–0.92]) (Table 1). Other institutional factors were not significantly associated with in-hospital mortality (Table 1). Regarding patient-based factors, elderly patients (40–59 years old 1.82 [1.03–3.20]; 60–79 5.21 [2.97–9.15]; ≥80 15.60 [7.23–33.66]), unfit patients (2.08 [1.44–3.02]), and patients who received a higher volume of initial FFP transfusion (>25 ml/kg 2.14 [1.23–3.73]) showed a significant association to higher in-hospital mortality. In contrast, female patients (0.72 [0.50–1.02]) showed a significant association to lower in-hospital mortality. Table 2 shows the relationship between hospital volume and therapeutic approach. The rate of immediate ATRA initiation was significantly higher in high-volume hospitals than in low-volume hospitals (83% vs. 76%, *p* = 0.004). Similarly, the rate of prompt initiation of conventional chemotherapy in addition to ATRA was significantly higher in high-volume hospitals (55% vs. 41%, *p* < 0.001).

**Table 1:** Multivariable logistic regression analysis with a generalized estimating equation for in-hospital death in all patients.

Variable		Patients (*n* = 1138)	Odds ratio	95% Confidence interval	*P* value
Institutional characteristics					
Center volume (patients/study period)	≤5	676 (59%)	Ref.	–	–
	>5	462 (41)	0.60	0.40–0.92	0.019
Hospitals in the urban city	No	720 (63)	Ref.	–	–
	Yes	418 (37)	0.94	0.62–1.43	0.788
Number of hospital beds	< 399	158 (14)	Ref.	–	–
	≥400	980 (86)	0.84	0.48–1.47	0.532
Educational hospital	No	45 (4)	Ref.	–	–
	Yes	1093 (96)	0.96	0.51–1.78	0.892
Hematologists per population in the prefecture	Tier 1: ≥3.200	339 (30)	Ref.	–	–
(/100,000 people)	Tier 2: ≥2.464, <3.200	338 (30)	1.03	0.63–1.67	0.907
	Tier 3: ≥1.950, <2.464	191 (17)	0.82	0.51–1.32	0.420
	Tier 4: <1.950	270 (24)	1.17	0.70–1.95	0.554
Patient characteristics					
Age, years	20–39	277 (24)	Ref.	–	–
	40–59	395 (35)	1.82	1.03–3.20	0.039
	60–79	371 (33)	5.21	2.97–9.15	<0.001
	≥ 80	58 (5)	15.6	7.23–33.66	<0.001
Sex	Male	582 (51)	Ref.	–	–
	Female	556 (49)	0.72	0.50–1.02	0.067
Body mass index, kg/m2	18.5–25	627 (55)	Ref.	–	–
	< 18.5	92 (8)	0.76	0.37–1.56	0.455
	25–30	306 (27)	0.68	0.43–1.06	0.090
	> 30	81 (7)	1.31	0.64–2.68	0.459
Activities of daily living	Fit	732 (64)	Ref.	–	–
	Unfit	292 (26)	2.08	1.44–3.02	<0.001
Charlson comorbidity index	2	960 (84)	Ref.	–	–
	≥ 3	178 (16)	1.13	0.73–1.77	0.580
Initial volume of fresh frozen plasma, ml/kg	< 9	267 (24)	Ref.	–	–
	9–25	580 (51)	1.06	0.64–1.76	0.817
	>25	271 (24)	2.14	1.23–3.73	0.007
Emergency admission	No	352 (31)	Ref.	–	–
	Yes	786 (69)	1.28	0.87–1.86	0.208

**Table 2:** Difference in therapeutic approaches between high- and low-volume centers.

		High-volume center (*n* = 462)	Low-volume center (*n* = 676)	*P* value
Initiation of all-trans retinoic acid	≤1 day after admission	384 (83%)	514 (76%)	0.004
	>1 day after admission	78 (17%)	162 (24%)	
Initiation of conventional chemotherapy	≤7 days after admission	253 (55)	274 (41)	<0.001
	>7 days after admission	108 (23)	221 (33)	
	Not performed	101 (22)	181 (27)	

Early death is also an important issue in daily practice in Japan, a developed country with a well-established universal health insurance system. This nationwide analysis suggest that high-volume hospitals may have benefited from a rapid decision-making process, thereby reducing lower mortality. Variation in early mortality between medical facilities in previous real-world studies raised the possibility that institutional factors could be determinants of early mortality in patients with newly diagnosed APL [16–19]. Our previous single-center study at the University of Tokyo Hospital reported preferable outcomes, and thus, we hypothesized that some of the institutional factors of the University of Tokyo Hospital may be important in preventing early death: a high-volume hospital, an urban hospital, an educational hospital, a hospital with a large number of beds, and a hospital in the prefecture with a large number of hematologists per population [20]. Among them, hospital volume was the only institutional factor that was significantly associated with early death in the current nationwide study.

To date, the impact of hospital volume on clinical outcomes had been mainly investigated in patients who received surgery [21–24]. Higher hospital volume has been reported to be associated with lower postoperative complications and favorable outcomes. In contrast, the relationship between hospital volume and the outcome of chemotherapy has not been fully elucidated. To our knowledge, only two studies from the United States reported the relationship between hospital volume and the prognosis of acute myeloid leukemia (AML) [25, 26]. These studies demonstrated that high-volume centers were significantly likely to be associated with a preferable prognosis of AML. However, previous studies focused only on hospital volume as an institutional factor, and other institutional factors were not evaluated. In this regard, our nationwide data showed that hospital volume was significantly associated with early mortality, independent of other institutional factors. It is important to note that because APL is a rare disease, hospitals were defined as high-volume if >5 (the median) patients were treated from 2010 to 2018. The current study also suggests possible reasons for preferable leukemia survival in high-volume hospitals, which has not been evaluated in previous studies; that is, high volume hospitals were likely to perform prompt and intensive therapeutic intervention.

Some study limitations must be considered. First, the database does not contain laboratory data or physical findings (e.g., plasma fibrinogen level). Thus, we could not evaluate peripheral white blood cell counts, which were reported to be a risk factor for early death. In addition, unrecognized fluid overload at the time of diagnosis could affect mortality [27, 28]. These physiological findings were also unavailable in this database. Second, the current study did not evaluate the effect of arsenic trioxide because it has not been approved for untreated APL in Japan. Finally, information on the cause of death was not included in the database. Major causes of early death in patients with untreated APL were reported to be hemorrhage due to coagulopathy, differentiation syndromes, and infection [13]. It is assumed that most early deaths in this study were also due to these causes; however, the actual cause of death could not be confirmed in this study.

In conclusion, treatments at a high-volume hospital are significantly associated with lower in-hospital mortality. Thus, in the real world, centralizing APL patients to high-volume hospitals may be beneficial for reducing early mortality.

## Supplementary information

Supplemental Table 1

## Data Availability

The database used in this study is not intended for public use.
