# Supplementary material for: Institutional factors associated with early mortality of newly diagnosed acute promyelocytic leukemia
Source: Blood Cancer J. 2022 Dec 15;12(12):167. doi: 10.1038/s41408-022-00767-6 (PMC9755312; doi:10.1038/s41408-022-00767-6)
Supplement: Supplementary file 1 — Supplemental Table 1 [file 41408_2022_767_MOESM1_ESM.docx]

**Supplemental Table 1** Hematologist per population in each prefecture in Japan

The 47 prefectures in Japan are equally divided into four tiers: tier 1: ≥3.200 (12 prefectures), tier 2: ≥2.464, <3.200 (12 prefectures); tier 3: ≥1.950, <2.464 (11 prefectures), and tier 4, <1.950 (12 prefectures).

**Number of hematologists per 100,000 people**

| Tier 1: ≥3.200 | | Hokkaido, Akita, Gunma, Tokyo, Ishikawa, Fukui, Kyoto, Mie, Okayama, Tokushima, Kagawa, Saga |  |
| --- | --- | --- | --- |
| Tier 2: ≥2.464, <3.200 | | Niigata, Tochigui, Aichi, Shiga, Nara, Osaka, Shimane, Kochi, Ehime, Fukuoka, Nagasaki, Kumamoto |  |
| Tier 3: ≥1.950, <2.464 | | Fukushima, Kanagawa, Nagano, Yamanashi, Toyama, Hyogo, Wakayama, Tottori, Yamaguchi, Oita, Kagoshima | |
| Tier 4: <1.950 | | Aomori, Iwate, Miyagi, Yamagata, Ibaraki, Saitama, Chiba, Shizuoka, Gifu, Hiroshima, Miyazaki, Okinawa | |
|  |  |  |  |
|  |  |  |  |
